# New Hybrid Algorithms for Estimating Tree Stem Diameters at Breast Height Using a Two Dimensional Terrestrial Laser Scanner

**DOI:** 10.3390/s150715661

**Published:** 2015-07-02

**Authors:** Jianlei Kong, Xiaokang Ding, Jinhao Liu, Lei Yan, Jianli Wang

**Affiliations:** School of Technology, Beijing Forestry University, Beijing 100083, China; E-Mails: kongjianlei_slgc@163.com (J.K.); dingxiaokang1985@126.com (X.D.); jianli.bjfu@163.com (J.W.)

**Keywords:** hybrid circle fit algorithms, diameter at breast height estimation, two-dimensional terrestrial laser scanner

## Abstract

In this paper, a new algorithm to improve the accuracy of estimating diameter at breast height (DBH) for tree trunks in forest areas is proposed. First, the information is collected by a two-dimensional terrestrial laser scanner (2DTLS), which emits laser pulses to generate a point cloud. After extraction and filtration, the laser point clusters of the trunks are obtained, which are optimized by an arithmetic means method. Then, an algebraic circle fitting algorithm in polar form is non-linearly optimized by the Levenberg-Marquardt method to form a new hybrid algorithm, which is used to acquire the diameters and positions of the trees. Compared with previous works, this proposed method improves the accuracy of diameter estimation of trees significantly and effectively reduces the calculation time. Moreover, the experimental results indicate that this method is stable and suitable for the most challenging conditions, which has practical significance in improving the operating efficiency of forest harvester and reducing the risk of causing accidents.

## 1. Introduction

In forestry, Light Detection and Ranging (LiDAR) devices are often used for remote sensing applications to record inventory parameters that can describe the state of forests. Besides traditional satellite laser scanning (e.g., ICESat-GLAS) [1,2] and airborne laser scanning (e.g., SLICER, LVIS) [3,4], small and portable terrestrial laser scanning (TLS) devices, which can be mounted on a static tripod or transported by a forestry vehicle, have been readily introduced into the field [5]. For forestry resource survey purposes, TLS has generally been a fast, efficient and automatic tool for determining basic properties and structural parameters of forests such as discrimination of plant components [6], stem count density [7], three-dimensional forest reconstruction [8], canopy height modelling [9], chlorophyll level measurement [10] as well as variables recognition [11]. Especially, the living-tree diameter at breast height (DBH) is the basic parameter in forestry resources surveys [12] and is a good predictor for many features of interest (e.g., above-ground biomass) [13,14]. Measuring the diameter of tree trunks is inherently related to the location and regeneration of the living trees and obstacles [15], thus a solution strategy to the advanced measurement and perception system for enhancing the automation of forestry vehicles such as forest harvesters [16]. A typically application is a forestry machine carrying a 2D laser scanner using simultaneous localization and mapping (SLAM) algorithms to create local tree maps of the environment in real-time [17,18]. The resulting maps, which are based on the estimated position and diameter of the trees, would be used for autonomous navigation, including path planning and obstacle avoidance [19]. This will improve various semi-autonomous functions including positioning the harvester head and selecting trees automatically, thus relieving the pressure of the operator [20,21]. In other words, DBH is important in the forestry area both for traditional remote sensing applications and advanced automation solutions.

A large number of experimental studies have confirmed the potential of TLS to successfully extract the DBH as mentioned. All of these have investigated the tree trunk diameter with three-dimensional terrestrial laser scanners (e.g., Faro LS 800, Riegl LMSZ420i, Leica HDS6000). As DBH is defined as the diameter 1.3 m above the finished grade at the end of the trunk, a horizontal slice with a thickness at a height of 1.3 m above the representative ground point is cut from a high resolution 3D point cloud in the usage of 3D terrestrial laser scanners. Then an adjusting circle is fit into the 2D projection of the points of that slice to estimate the DBH as well as location, and high level tree features [7,12]. However, 3D scanners are expensive and impose hardware limitations in laser data processing, which makes them unsuitable for the real-time diameter-measuring equipment used by forestry harvesters and their autonomous navigation. In the experiments on forestry harvesters, the processes of feeling, delimbing, peeling, and cutting-in-length can be completed fast, but during the process of the alignment of the harvesting head to capture the trunk, the operator has to perform repeated observations, judgments and operations due to the complex forestry environment and the continual vibration of the vehicle, which lead to time and fuel losses and reduce the logging efficiency [22]. Therefore, it is significant to find an efficient and fast method to confirm target trees for the harvesting head to achieve automatic capture. This leads to the fact that low-cost and robust 2D terrestrial laser scanners has been successfully used to obtain the point cloud of the surrounding trees for a better price-performance ratio in logging operations. For estimating the diameter information of tree trunks from TLS data accurately, a large number of related studies have been carried out.

A standard pattern recognition method with a Hough-transformation was applied by Aschoff and Spieker [23] to detect the trees and extract the features. The diameter of a tree is determined as an adjusted circle and adjusted ellipse. By using the circle fitting algorithm, better results were produced concerning their arithmetic mean and their maximum in comparison to the ellipse fitting. Therefore other researchers have looked beyond the ellipse mode, seeking efficient and high-accuracy circle fitting algorithms for estimating the DBH based on the 2D laser data. For instance, some apply geometric distances from the circle center to the detected cluster points to estimate the trunk diameter, which is defined as geometric fit [24]. The two triangle diameter estimation (TDE) is a fundamental geometric model for living-tree diameter estimation (STDE) in the application of 2D laser scanners. This method uses two right-angled triangles (TDE) to confirm the center point of the fitting circle and the tree trunk diameter, with the shortest range and angular resolution of the laser scanner in the original work [25]. To improve the accuracy of the measuring system, the first and the last cluster with the resolution of the laser scanner based on viewing angle were proposed by Jorma to estimate trunk diameter [26].

Others methods have been developed to match laser data as circles for estimating a trunk diameter. Those geometric fitting approaches aim at minimizing the error between the sum of the squares of the distances of laser points and the radius of the fitted circle. There exist various numerical algorithms to find the circle that best fits a given set of measured laser data pairs. The problem of solving the equation of a circle is restated by Wang [27] as a linear least square fitting (LST) problem, which estimates the DBH quickly and accurately. In order to improve the time consumption of calculations compared with the LSF algorithm, the Fletcher-Reeves conjugate gradient algorithm (FR) is applied to calculate the radius and center locations of the trunks in the scanning range. This method was validated through experiments of automatic trunk capture for the harvesting head [28]. Similarly, the Polak-Ribiere-Polyak conjugate gradient algorithm (PRP) is also described as a circle fitting procedure for 2D laser point clouds. The method meets the requirements for the automatic selective cutting of the logging harvester [29].

However, a major concern in geometric fitting is that the minimization algorithms require iterative and computationally intensive numeric schemes. Thus the algorithm estimating the DBH fits an algebraic equation to represent a circle. Corresponding algebraic fitting methods such as the Kasa algorithm are non-iterative and thus faster than geometric fitting as reported in [30]. Here the residual tree stems in the layers are mapped as circle rings, which are detected by using a Hough-transformation method and fitted as circles accurately by using the Kasa algorithm. On the other hand, it has been found that the accuracy of the Kasa fitting suffers in cases when the observed points do not represent complete circular arcs [31]. Thus, several modifications have been developed to overcome this limitation in single scan mode. The algebraic-based algorithms Pratt and Taubin are tested to circle fit the DBH accounting for the incomplete laser circular representation [32].

Nevertheless, their performances strongly depend, among other factors, on the choice of the initial laser data of the 2D laser scanner (2DLS). When a single 2DLS laser pulse is sent out and reflected by an object surface within the range of the scanner, the elapsed time between emission and reception of the laser pulse serves to calculate the distance between the object and the 2DLS [33]. Since the reflectivity is based on the material and position of the various objects, a ranging error exists in a forest raw point clouds, which causes a loss of accuracy in estimating the DBH severely in circle fitting. Therefore, robust estimation techniques allow gross errors in the data points to be eliminated, which may for instance be caused by leaves, twigs, neighbouring shrubs or instrument errors, thus warranting high reliability of the measuring system. Since a laser scanner typically generates several scans per second with a frequency of around 50 Hz, it is possible to combine multiple scans even for mobile applications. In consideration of the laser scanning data error caused by the laser beam width, the circle fit algorithms combined with beam width compensation by fusing multiple scans (CFAA-MS) was chosen to modify outer points and all tree cluster points in order to adjusting the diameter estimation [34,35].

However, a systematic study of the factors which influence the accuracy of information extracted from laser data DBH estimation algorithms is still lacking, even though the need for such analyses is already formulated at quite an early stage. To solve this problem, this paper investigates the possibility of using enhanced algorithms with polar parameters to estimate the diameter of tree trunks by using 2DLS data. This work applies the algebraic fit method to linearly fit the discretely distributed laser points as a circle to form the initial guess. Then the Levenberg-Marquardt scheme is selected to minimize the algebraic distances from the contour points to the resulting circle nonlinearly. After cluster extraction and filtering, this hybrid algorithm uses the arithmetic mean method based on multiple scans to adjust the original laser points for obtaining a higher accuracy in the diameter estimation. From the comparison results, this proposed method improves the accuracy of DBH estimation and effectively reduces the calculation time, which is also affected weakly by the harsh environment puzzling the drivers and suitable for the challenging conditions in forestry.

The rest of this paper is as follows: Section 2 briefly addresses algorithms for 2D point cloud segmentation and a new algorithm to improve the accuracy of 2DLS. Section 3 presents the principle and notations of our new circle fit method for DBH. Compared with related methods, experimental results obtained in a number of pilot studies will be analyzed in Section 4. Lastly, Section 5 concludes the algorithm presentation in this paper.

## 2. Experimental Description and Trunks Feature Extraction

### 2.1. The Experimental Facilities

For continuous accurate measurements rapidly, a LMS511-pro type 2D laser scanner produced by the SICK Company (Waldkirch, Germany) is used as the essential sensor to build the system for measuring the DBH parameter of living-trees in forest areas. The measurement data corresponding to the surrounding contour scanned by the LMS511-pro is output in hexadecimal format to form the raw point cloud via the Ethernet interface at the rate of 100 Hz. A computer having a conventional Windows 7 operating system installed is applied to link with the 2D laser scanner and analyze the measurement data, which are stored exclusively for post-processing. In the PC, the actual data acquisition and analytical software is programmed with M language to set the operating parameters of the laser scanner and handle the laser data in offline model by using Matlab 2012b.

**Figure 1:**
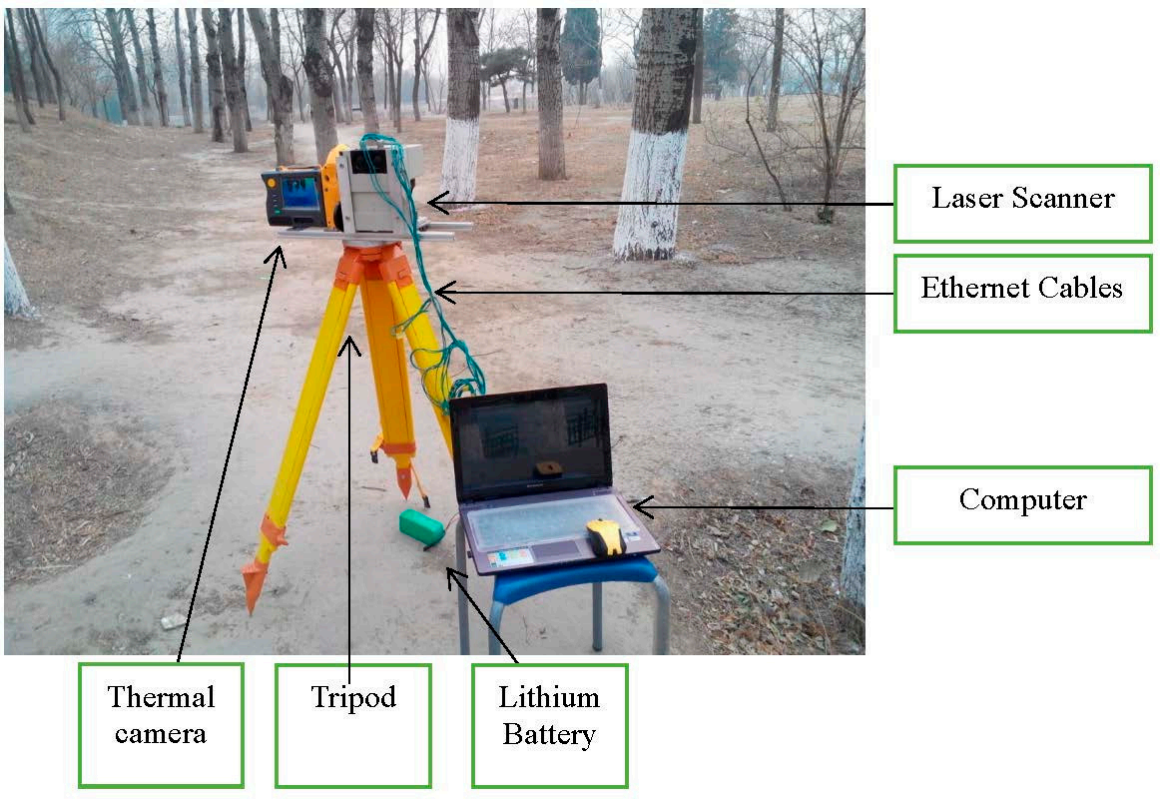
The measurement equipment includes sensor equipment fixed on the tripod platform as well as the data acquisition PC and lithium batteries providing 24 V for the system. The experiment is used in a birch forest.

To acquire abundant tree features with adequate resolution from the laser cloud measurements taken in the forest, the scanning angular resolution of the LMS511-pro is set to its minimum value 0.1667°. Then the maximum scanning angle is set to 100° and maximum scanning distance is 32 m. Therefore, the measurement result of LMS511-pro laser scanner is a right ahead semicircle in the front of the device, while its centre is the scanner’s location, the radius is 32 m and the scanning degree is 100° in a range from 40° to 140°. The electronics of the LMS511-pro are directly powered by a 24 V lithium battery. In order to recording the corresponding visual information, a Fluke TI55 type infrared thermal camera with images of 640 × 480 pixels resolution is mounted on the side of the laser scanner. This device can obtain both RGB visible and infrared thermal images simultaneously and regularly. Visible images reflect the visual reality and infrared thermal images record the temperature of the environment. Because of the complicated surroundings in a forest, it is difficult to detect targets guided by any single information source. When it is dark or misty in the forest, it becomes difficult to distinguish the objects in the RGB images without information about temperature, whereas after a period of extensive cooling (e.g., after a long period of rain or early in the morning), the infrared images are less detailed in representing the background due to the low thermal levels compared with visible images. In this situation, the fusion of the visible and thermal image on a single display could enhance the fused images’ clarity and capture more abundant information about the reality. Therefore, an algorithm based on a Contourlet transform and a pulse coupled neural network (PCNN) is used to generate the mutual complementary blending images (the detail description can be found in [36]). Considering that the outdoor experiments are carried out in different places, this paper will apply those fused images to track both the pose of the laser scanner and the measured trees, which will establish a corresponding relationship between the fitted diameter data and the corresponding observed diameter for further accurate data processing. The complete measurement equipment setup is shown in Figure 1.

**Figure 2:**
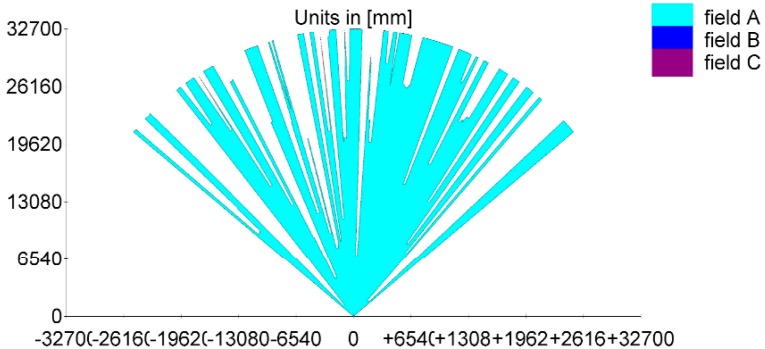
The raw laser data scanning result.

**Figure 3:**
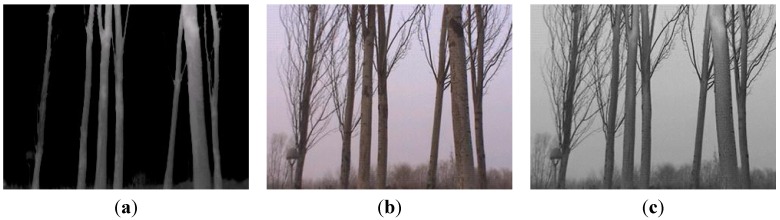
The corresponding visual information of the forest area: a visible image (**a**); an infrared thermal image (**b**); a fused image with clarity enhanced and more abundant captured information (**c**).

To measure the DBH of living-trees in a real forestry environment, outdoor experiments were carried out in birch forest located in the Peking Olympic Park. In our experiments, the 2D laser scanner is fixed on a tripod with telescopic legs as seen in Figure 1. The laser scanner sends and receives the laser beams reflected by tree trunks or other objects to form a fan-shaped scan, which is represents the surrounding area as shown in Figure 2. Here the height of the scanning plane is equal to about 1.3 m from the ground, as measured by a tape. This leads to better results because the understory and other uninteresting objects lie below the scanning plane in this experiment. The equipment was placed at different 15 places and 60 trees were nearly totally scanned. In order to completely demonstrate the procedure of laser data optimization and DBH estimation, only the raw laser clouds at the position with the eight silver birches are presented in the experiment results using real data. Those targets are applied to reveal the DBH estimation error fitted by the hybrid algorithm in contrast with the manual work. The actual laser data is shown in the Figure 2, and the images obtained by the thermal camera are seen in Figure 3. However, all 60 targets measured are used to confirm the excellence of the proposed method compared with other algorithms in the comparison part.

**Figure 4:**
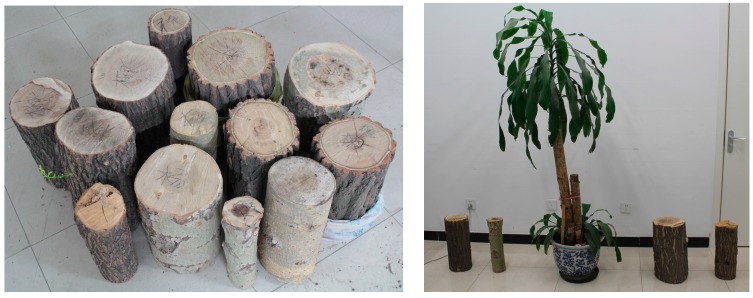
The thirteen tree trunks and the indoor experimental scene.

In order to increase the target quantity, a further outside and indoor simulation is performed to reveal the influence of the distance and diameter on the diameter estimation error. For each abovementioned birch, the 2DTLS scanned all eight targets at a distance ranging from 2 m to 12.2 m every 0.6 m to the tree. Then, thirteen tree trunk sections with diameters in the range of 9–35 cm and lengths in the range of 40–49 cm are used in the indoor experiment. They are also placed at distances from the 2DTLS varying between 2 m and 12.1 m in 0.3 m steps. The diameter range and length of the trunks are recorded in Figure 4 and Table 1. In total, 378 sets of laser data integrating the outside and indoor measurement experiment are scanned to calculate the diameter error for a better statistical consequence.

**Table 1:** Tree species, diameter range and length of the trunk sections scanned in the experiment.

Species	Diameter Range (mm)	Length (mm)
Weeping willow (*Salix babylonica*)	173.6–178.2	470.8
Weeping willow (*Salix babylonica*)	264.2–279.4	401.4
Weeping willow (*Salix babylonica*)	310.9–347.5	433.2
Weeping willow (*Salix babylonica*)	243.2–250.4	427.1
False acacia (*Robinia pseudoacacia*)	225.8–227.2	429.6
False acacia (*Robinia pseudoacacia*)	147.8–148.4	452.4
False acacia (*Robinia pseudoacacia*)	296.2–310.5	416.1
False acacia (*Robinia pseudoacacia*)	235.8–256.4	482.6
Abele (*Populus alba*)	259.0–266.2	411.5
Abele (*Populus alba*)	158.4–160.8	416.1
Abele (*Populus alba*)	197.2–198.1	420.5
Abele (*Populus alba*)	294.4–316.1	429.4
Silver birch (*Betula platyphylla*)	99.4–101.8	408.8

### 2.2. Clustering, Filtering and Extracting the Trunks

**Figure 5:**
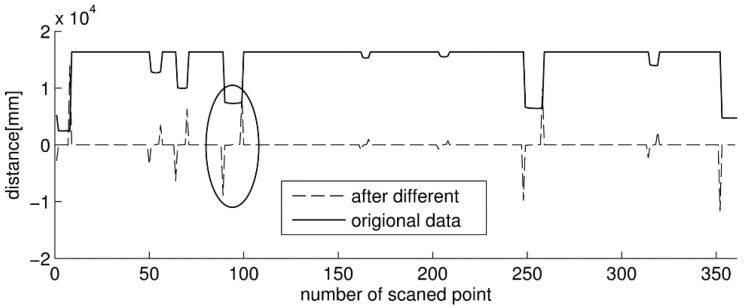
The schematic of the difference calculation for clustering the raw laser data.

As the measurement data is processed in increasing order of the bearing angle from 40° to 140° with the chosen angular resolution, a vector *L_i_* = [*D_i_*, θ*_i_*] in polar form is supposed to describe the single laser beam, where *i* is the sequence of laser beam distribution from 1 to 601, θ*_i_* is the angular position and *D_i_* is the value of the horizontal distance between the laser reflecting point and the device. Before filtering, the raw laser data are passed through the clustering algorithm based on difference calculation to form a difference vector satisfying the following equation:

(1) ΔD(i)=D(i+1)−D(i)

where ∆*D*(*i*) is the new difference vector representing the range between two adjacent laser beams *L_i_* and *L_i_*_+1_. As shown in Figure 5, dramatic changes will occur in the value of the difference vector when the edges of objects are detected in the original laser data [37]. After calculation of the difference, the independent objects are separated from the background in the vector curve as follows.

Then the values of vector are compared with the depth threshold ∆*R_max_*, which is the allowed difference in ranges inside a cluster. If two neighbouring differences ∆*D*(*m*) and ∆*D*(*n*) satisfied the constraint as follows, the laser cloud clusters will be selected for further disposition:

(2) $$\text{Δ}D(m)>\text{Δ}R_{\max};\text{Δ}D(n)<-\text{Δ}R_{max};N_{min}<n-m<N_{max}$$

where *N_min_* is the smallest acceptable width of the cluster and *N_max_* is the related greatest acceptable width of the cluster. Those laser points from the *m*-th to the *n*-th belong to the cluster and the others belongs to the background or another cluster. 

Using Equation (2) is certainly effective and sufficient for extracting laser clusters of targeted trunks from the point cloud in a forest with a few bare living-trees. However, the laser beam may be reflected by uninteresting things such as branches, stones or the ground in a more complex environment, which causes measurement errors in the point cloud. Thereby, the incorrect points clustered from the raw laser cloud should be filtered out with some detective criteria to obtain the actual trunk clusters. Here the filtering is performed by testing the curvature of each cluster, which describes the diameter of the tree. If the feature width and the curvature inspection satisfy the constraints simultaneously, the clusters will be accepted as trunk features of living trees. The curvature values are calculated for each individual point *l*(*j*) (*m* < *j* < *n*) that has surrounding points as follows:

(3) l(j)=D(j+1)+D(j-1)-2D(j)

By using the curvatures of each point, the curvature of the whole cluster is calculated as follows:

(4) $$curv=\frac{\sum_{j=m}^{n}l(j)}{(n-m)L_{min}^{2}}$$

**Figure 6:**
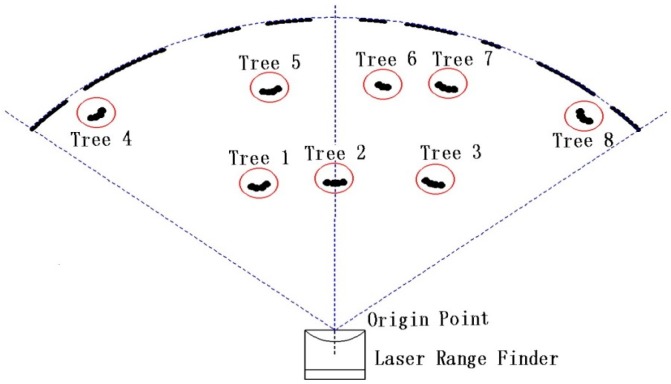
The clustering and filtering result of the experiment in polar form.

In the equation, *L_min_* is the minimum measured range of the cluster and *j* is the sequence of points in the cluster. For both the individual points and the whole cluster, the values must be greater than or equal to zero, which means the laser clusters all have a convex surface. Moreover, the measurement clusters also satisfy following criteria:

(5) $$l(j)\geq 0;curv\geq 0;curv_{min}<curv<curv_{max}$$

where *curv_min_* is the minimum curvature of the whole cluster and *curv_max_* is the corresponding maximum limit, which prescribes the acceptable value scope of the trunk radius. Finally the laser data clustered by the acceptable width and depth in Equation (2) can filter out the ground or other uninteresting things with the above Formula (5). Considering that the distance between two trunks is large in the experiment, ∆*R_max_* is chosen as 0.8 m and the width of cluster is limited in the range of 3 to 50 based on the divergence of the laser scanner. Eventually, eight clusters can be confirmed as tree trunks from the raw point cloud after clustering and filtering as shown in Figure 6.

### 2.3. Laser Point Cloud Optimization

According to the working principle of 2D laser scanners, the distance value of a laser beam is influenced by the reflectance of objects and the returned energy of the laser beam. In the actual process of a single continuous measurement, the laser reflection ability of various objects is not only directly affected by the coarseness and color of objects, but also influenced by the incidence angle and laser beam spot size.

With the discrepancy of reflectivity and the change of the laser speckle, the size of the laser energy obtained by the measurement instrument is different in the same laser beam as time goes by. Therefore, the measuring value of the same laser beam are also disturbed by fluctuating errors like the temporal extension. This is the main factor affecting the measuring accuracy of a laser scanner in the application for DBH estimation. To confirm the range of the error, a white board is located in 2 m distance from center of the laser scanner and perpendicular to the 24-th laser beam. The measured distances for 100 scans are obtained to establish the relationship between multiple scans of an object and the corresponding frequency of occurrence as follows.

**Figure 7:**
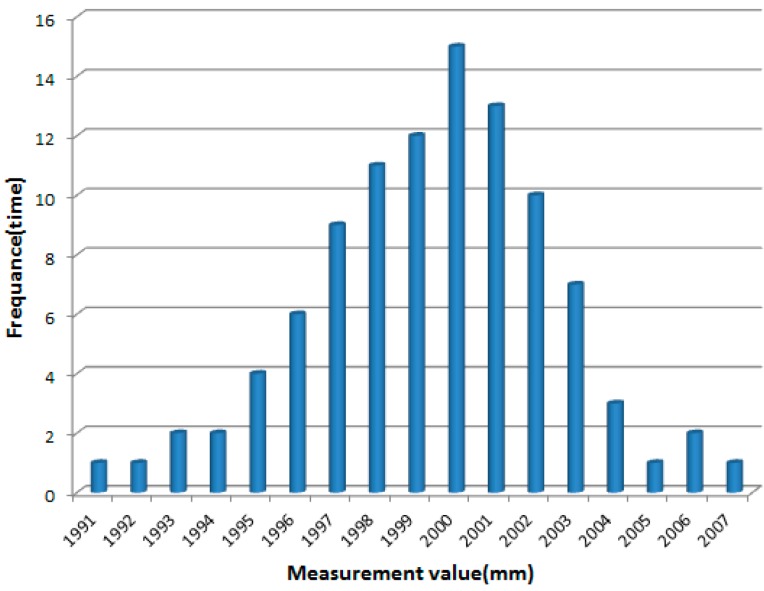
Relation histogram of the measured values *vs.* to the corresponding frequency.

As shown in Figure 7, the result indicates that the experimental distance values are normally distributed around a measurement value of 2000 mm approximately, which is equal to the actual distance between the object and the 2DTLS. Since the error is distributed in different regions in the range of plus or minus 10 mm, the 95-percent confidence interval of this normal distribution of the fit is plotted in the histogram as follows:

(6) $$\bar{m}-3\xi <P_{i}<\bar{m}+3\xi$$

where the initial parameter values *P_i_* represents the distant value of the *i*-th laser point measured by 2DTLS. The mean or expectation of the distribution $\bar{m}$ is confirm as $\bar{m}=2000$ and its standard deviation is ξ = 2. With the correlation matrix of the fit parameters being tabulated, the true distance values lie within the confidence interval with a confidence level of 95 percent. Considering that the expectation of multiple scanning data is approximate to the desired measuring data, the mean of the distance value of the same laser beam at different moments represents the expectation of the standard normal distribution, which is applied to reduce the fluctuating error. Thus the arithmetic mean method is applied to calculate the distance mean value, which is close to the actual distance value compared with the distance value of one single scanning datum at a random moment. *N* consecutive samples values are arithmetically averaged with mathematical expression as follows:

(7) $$\bar{P}=\frac{1}{N}\sum_{i=1}^{N}P_{i}$$

where *N* is the number of scan times and $\bar{P}$ is the arithmetic mean after optimizing. This algorithm reduces the fluctuant error of laser scanner data and promotes the initial raw laser data in a certain degree. With the noisy optimization of all laser beams, the estimation of the trunk diameter with the mean of several repeated laser scans is better than independent single estimates. 

## 3. Method of Hybrid Circle Fit

After extracting and optimizing the trunk features from the point cloud, the trunk clusters are ready for the DBH estimation with the circle fitting algorithm. There exist a number of different methods to fit a circle and estimate its parameters [38]. However the best fitting arc of traditional methods depend on a Cartesian coordinates system. This is not suitable for measuring the laser scanner clouds, which are obtained originally in polar form. If the raw data points changed to the Cartesian coordinate form lie along a circular arc with low curvature, the best fitting circle would have a large radius R and a far deflected center, which do not fit the real situation. To avoid this problem, this paper uses the polar form to calculate the so-called algebraic circle fitting parameters.

Another issue is that the accuracy of diameter estimation in the mentioned studies is severely influenced by the initial guess. If the initial guess is picked at random or disturbed by noise, the chance of divergence may be very high [39]. To resolve this issue, a new circle fitting algorithm is proposed to calculate the DBH in two steps. Firstly, the initial parameters of the fitted circle are confirmed by a non-iterative algebraic operation in polar form. Then the geometric distances from the measured points to the fitting circle are minimized to fix the initial guess in the Levenberg-Marquardt method with a modified convergence principle, which generally eliminates the non-linear errors [40].

For estimating the diameter with the new hybrid method, it is necessary to assume that the cross section of the living-tree is an ideal circle and there are at least three laser points in polar form located on the living-tree. 

Similarly, we assume that the vector *P_i_* = (*l_i_*cosα, *l_i_*sinα) represents the position of every point in the trunk cluster in polar form, where *l_i_* is the distance value of the *i*-th measurement and α is the corresponding azimuthal angle. Supposing that the expression *O*(*O_x_*, *O_y_*) = (ρcosθ, ρsinθ) represents the center of the fitting circle and its radius is assumed to be *R*, here (ρ, θ) is the distance and related angle of the circle center. Then, the vector *P_i_* satisfies the fitting circle with polar coordinate as follows:

(8) $$R^{2}={\left(l_{i}\cos\alpha_{i}-\rho •\cos\text{θ}\right)}^{2}+{\left(l_{i}\sin\alpha_{i}-\rho •\sin\text{θ}\right)}^{2}$$

To obtain an initial guess for the circle center, the cost function *E* was minimized as follows:

(9) $$E(\rho ,\text{θ},R)=\sum_{i\geq 3}^{n}\left\|{l_{i}}^{2}+\rho^{2}-2l_{i}\rho \cos(\alpha_{i}-\text{θ})-R^{2}\right\|\to \min$$

This equation can be solved by setting:

(10) $$\frac{\partial E}{\partial \rho }=\sum_{i\geq 3}^{n}\left\|2\rho -2l_{i}\cos(\alpha_{i}-\text{θ})\right\|=0;\frac{\partial E}{\partial \text{θ}}=\sum_{i\geq 3}^{n}\left\|2\rho l_{i}\sin(\alpha_{i}-\text{θ})\right\|=0$$

Simplify Formula (9) to get the initial parameters of the fitting circle:

(11) $$\begin{array}{l} {\text{θ}}_{0}=\arctan(\frac{\sum_{i\geq 3}^{n}\left\|l_{i}\sin\alpha_{i}\right\|}{\sum_{i\geq 3}^{n}\left\|l_{i}\cos\alpha_{i}\right\|});\rho_{0}=\frac{\sum_{i\geq 3}^{n}\left\|l_{i}\cos(\alpha_{i}-\text{θ})\right\|}{n-2};\\ R_{0}=\frac{1}{n-2}\sum_{i\geq 3}^{n}\left\|{l_{i}}^{2}+\rho^{2}-2l_{i}\rho \cos(\alpha_{i}-\text{θ})\right\| \end{array}$$

Once the initial guess for the circle center is ensured, we need to improve the circle for some definition of best fit against the points set. Using an iterative method for nonlinear least squares problems such as the Levenberg-Marquardt estimator based on the geometry distance between the points and the circle is a wise choice [41].

To improve the circle fitting with an independent variable *x* of *m* parameters to a set of *n* data points *t_i_* = (*l_i_*, *a_i_*), it is customary and convenient to minimize a given function *F*(*x*), which presents the sum of the weighted squares of the errors between the measured data *d_i_* = (*x*, *t_i_*) and the curve-fit radius *R*:

(12) $$F(x,t_{i})=\sum_{i\text{=}1}^{n}{\left\|d_{i}-R_{0}\right\|}^{2}\to \min$$

where *x* = (ρ, θ, *R*) and $d_{i}=\sqrt{{\left(l_{i}\cos\alpha_{i}-\rho •\cos\text{θ}\right)}^{2}+{\left(l_{i}\sin\alpha_{i}-\rho •\sin\text{θ}\right)}^{2}}$. To simplify the target function, a derived function $f(x,t_{i})=\left\|d_{i}-R_{0}\right\|$ is given to change the equation as follows:

(13) $$F(x)=\frac{1}{2}\sum_{i\text{=}1}^{n}f_{i}{\left(x,t_{i}\right)}^{2}=\frac{1}{2}{\left\|f(x)\right\|}^{2}=\frac{1}{2}f{\left(x\right)}^{T}f(x)\to \min$$

Thus this general optimization problem can be solved by finding *x_min_* to minimize *f*(*x*) equivalently:

(14) $$x_{min}=\arg\min\left[F(x)\right]$$

Providing that the function *F* is differentiable and *f* has continuous second partial derivatives, the function evaluated with perturbed model parameters may be locally approximated through a second-order Taylor series expansion as follows:

(15) $$f(x_{\min}+h)=f(x_{\min})+J(x)h+O({\left\|h\right\|}^{2})$$

where *h* is a random perturbation, and $O({\left\|h\right\|}^{2})$ is the sufficiently small term which can be omitted. *J*(*x*) is the Jacobian matrix that contains the first partial derivatives of the function components as follows:

(16) $$J(x)\text{=}\frac{\partial f(x)}{\partial x}=\left[\begin{array}{ccc} \frac{\partial f(x,t_{1})}{\partial \rho } & \frac{\partial f(x,t_{1})}{\partial \text{θ}} & \frac{\partial f(x,t_{1})}{\partial R}\\ \vdots & \vdots & \vdots \\ \frac{\partial f(x,t_{n})}{\partial \rho } & \frac{\partial f(x,t_{n})}{\partial \text{θ}} & \frac{\partial f(x,t_{n})}{\partial R} \end{array}\right]$$

As regards *F*, its partial differential is expressed as:

(17) $$F^{'}(x)=\frac{\partial F(x)}{\partial x}\text{=}\sum_{i\geq 3}^{n}f_{i}(x)\frac{\partial f_{i}(x)}{\partial x}=J{\left(x\right)}^{T}f(x)$$

Similarly the Hessian of *F* in position (*j*, *k*) is:

(18) $$H(x)=F^{''}(x,t_{i})=\sum_{i\geq r}^{n}\left[\frac{\partial f_{i}(x)}{\partial x_{j}}•\frac{\partial f_{i}(x)}{\partial x_{k}}+f_{i}(x)\frac{\partial^{2}f_{i}(x)}{\partial x_{j}\partial x_{k}}\right]\text{=}J{\left(x\right)}^{T}J(x)+\sum_{i=1}^{n}f_{i}(x)f_{i}^{''}(x)$$

This shows that *F* is approximately quadratic in the perturbation *h*, finding the perturbation *h_lm_* to minimize the function *F* as:

(19) $$\frac{\partial F(x_{\min}+h)}{\partial h}\approx \text{-2}f{\left(x_{\min}\right)}^{T}J(x)+2h^{T}J{\left(x\right)}^{T}J(x)=0$$

The resulting normal equations for the Levenberg-Marquardt perturbation are:

(20) $$\left[J{\left(x\right)}^{T}J(x)+\text{μ}I\right]h_{lm}=-g,with\;g=J{\left(x\right)}^{T}f(x),\text{μ}>0$$

where small values of the algorithmic parameter μ result in a Gauss-Newton update and large values of μ result in a gradient descent update. The parameter μ is initialized to be large. If an iteration happens to result in a worse approximation, μ is increased. As the solution approaches the minimum, μ is decreased, the Levenberg-Marquardt method approaches the Gauss-Newton method, and the solution typically converges rapidly to the local minimum.

The stopping criteria for the algorithm should reflect that at a global minimizer, thus the LM algorithm terminates when at least one of the following conditions is met:

(1) The magnitude of the gradient of *J^T^*(x)*f*(x) drops below a threshold δ_1_: $\left|J^{T}(x)f(x)\right|\leq \delta_{1}$
(2) The error *f*(x)*^T^f*(x) drops below a threshold δ_2_: $\left|\frac{f{\left(x\right)}^{T}f(x)}{n-m+1}\right|\leq \delta_{2}$
(3) The relative change in the magnitude of *h_lm_* drops below a threshold δ_3_: $\left|h_{i}/x_{i}\right|\leq \delta_{3}$
(4) A maximum number of iterations *K*_max_ is completed safeguard against an infinite loop: K ≥ *K*_max_.

Otherwise, iterations terminate when the iteration count exceeds a pre-specified limit. In our experiment, *K*_max_ is set to 1000 and the initial threshold δ_1_ = δ_2_ = 10^−4^, δ_3_ = 10^−5^ consequently, faster convergence can be expected. The optimized estimation of the DBH and other trunks’ parameters in the horizontal plane can be calculated via this hybrid circle fit algorithm. For detailed explanations of the LM method, readers should refer to [42]. 

**Figure 8:**
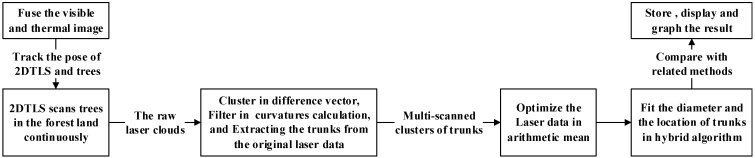
The analysis flow of DBH estimation with 2DTLS data for the harvesting head.

Finally, the overall data analysis flow of the equipment for the measurement and calculation of the tree parameters is shown in Figure 8. It is mainly divided into six consecutive phases. The first phase is fusing the visible and thermal image to track the pose of 2DTLS and the trees. Then 2DTLS scans the trees in the forest area continuously and projects the raw point cloud onto a horizontal scanning plane according to the angle resolution. The third phase is clustering trunks in difference vectors and filtering the invalid scanning data against some criteria of curvatures calculation, then extracting each trunk from the calibrated point cloud. Furthermore, those multi-scanned laser data are optimized in an arithmetic mean algorithm for reducing the fluctuating errors of the laser scanner data. The fifth phrase is determining the trunk diameter and location of the trunks in the proposed method for the harvesting head. Lastly, compared with related works, the sixth phrase is storing and displaying the results and graphing the useful information on the human-computer interface.

## 4. Experimental Results and Discussion

### 4.1. Experiment Results with Real Data

The trunk feature extraction process presented in the previous section was programmed in MATLAB. All of the calculation results such as radius, location of the trunks and distances between adjacent trunks could be displayed on a human-computer interface for the researchers’ use. 

**Figure 9:**
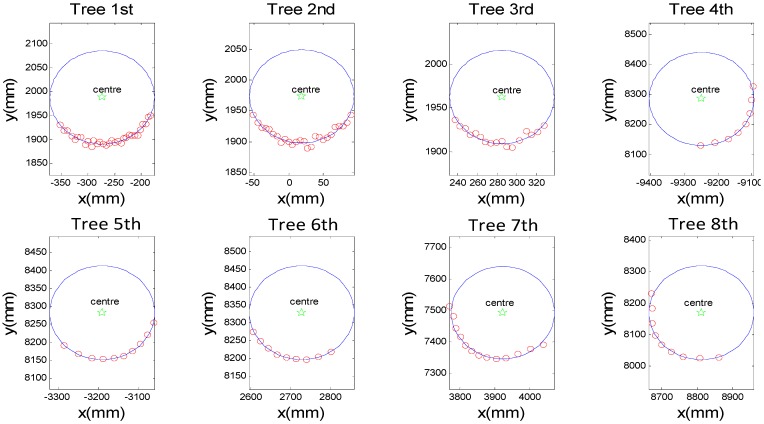
The fitting results of the trunk with laser clouds acquired by 2DTLS.

In the experiment, there were eight fitted circles representing living-trees chosen to estimate the trunk parameters in this proposed method in contrast with manual work. Supposing the measurement base point the origin of laser scanner in the polar form, the fitting results of the trunk point cloud (red points) are shown as the blue circles in Figure 9 and the centres of the circles were marked by the green five-pointed stars.

The parameters of the trunk could be extracted from the point cloud with the new fitting algorithm, as shown in Table 2. In addition, several contrast parameters were also selected to verify the high accuracy of the new algorithm proposed in this paper as shown in Table 2. 

**Table 2:** Parameters of the trunks acquired by different methods and the corresponding error.

Sample	Manual		New Algorithm		Error			
	Center Location (mm)	Radius (mm)	Center Location (mm)	Radius (mm)	Central Angular Deflection (degree)	Central Distance Deflection (mm)	Radial Absolute Error (mm)	Radial Relative Error (%)
Tree first	(−275.4, 1986.1)	96.89	(−273.5, 1987.9)	97.611	0.061	1.557	0.721	0.744
Tree second	(20.2, 1984.3)	78.21	(18.6, 1973.5)	75.534	0.043	10.829	2.676	3.421
Tree third	(288.6, 1990.6)	54.22	(285.3, 1972.6)	53.233	0.00057	4.276	0.987	1.821
Tree fourth	(−9260.3, 8302.7)	146.315	(−9249.3, 8285.7)	155.331	0.024	19.562	9.016	6.162
Tree fifth	(−3200.5, 8282.5)	134.205	(−3190.5, 8282.5)	130.082	0.060	3.604	4.123	3.072
Tree sixth	(2722.9, 8335.1)	127.61	(2727.7, 8328.0)	131.496	0.044	5.307	3.886	3.045
Tree seventh	(3913.9, 7502.5)	143.82	(3922.9, 7492.7)	146.877	0.084	4.544	3.057	2.126
Tree eighth	(8802.7, 8172.0)	156.52	(8812.3, 8170.1)	148.545	0.038	5.709	7.976	5.096

The manual measurement values of the central location and radius of the trunk were also given in the 1st and 2nd column of Table 2, which were acquired manually by a range finder and a Vernier, respectively. The error values were evaluated for systematic bias between the manual measurement centres and the estimated results via the hybrid algorithm in the 5th and 6th column, whereas, the 7th and 8th column gave the absolute error and as relative percentage error of radius to analyze if the diameter of the fitting circles was significantly separated from the manually measured value. Absolute error was calculated as the absolute value of the difference between estimated and observed diameter, while the relative percentage error was calculated as the absolute error divided by the observed diameter and then multiplied by 100. The errors were mainly caused by the resolution and systematic errors of the laser device and its related interaction effects of the new algorithm. The detailed results within all samples were shown as follows: as shown in Table 2, the angular deflections of the center locations were nearly the same for all targets, which were less than 0.1 degree, but the distance deflections of the center location were significantly increased as the distance between the base point of the laser scanner and the trunk increased. The maximum error of distance deflections was obtained for the fourth tree, which indicated that a stout tree with large diameter resulted in higher errors than a slender tree at the same distance. Moreover, the absolute error and the relative error of radius were also influenced by the trees’ real diameter and the distance to the tree. The max radial error of the calculation was less than 10 mm at nearly 13 m, which met the requirements of the accuracy for logging harvesting operations and other mobile applications in a forestry environment.

### 4.2. Comparison

To evaluate the effect of circle fitting optimization and beam improvement with multiple scans, the proposed algorithm was analyzed in a series of computer tests by comparing the results with those obtained with other methods. As competitors, two triangle diameter estimation (TDE) methods described in [23] was chosen. Then the circle fit algorithms combined with beam width compensation by fusing Multiple Scans (CFAA-MS) in [34] was also chosen to evaluate the diameter. Similarly, the least square fitting algorithm (LSF) in [26], Fletcher-Reeves conjugate gradient algorithm (FR) in [27] and the Polak-Ribiere-Polyak (PRP) conjugate gradient algorithm in [28] based on algebraic circle parameters were also selected to calculate the DBH. Here all 60 targets measured in 15 positions are used to estimate the parameters for confirming the excellence of the proposed method compared with other algorithms.

To verify the feasibility of the proposed circle detection algorithm, Gaussian noises with mean 0 and standard deviation 5 (mm) were also added to the trunk clusters in the radar slice plane detected by scanners, which were approximately the same as the observed noise distribution in sensors. The Gaussian noises were also independent among trials. Several contrast parameters were selected to verify high accuracy of diameter estimation with the new algorithm proposed in this paper as shown in Table 3.

**Table 3:** Experimental result of new algorithm compared with related works.

Parameter	TDE	LSF	F-R	PRP	CFAA-MS	New Method
Average radial absolute error (mm)	13.976	8.341	5.761	6.599	5.894	3.655
Max error of radius (mm)	26.089	13.078	11.469	12.716	10.805	8.579
Average radial relative error (%)	12.125	7.486	4.707	5.683	5.253	2.893
RMSE	15.671	8.913	6.748	7.277	6.465	4.464
STD	7.580	3.358	3.757	3.277	2.839	2.742
R-square	0.161	0.729	0.844	0.819	0.857	0.932
Time consumption (ms)	0. 019	6.296	488.622	288.789	9.771	4.745

As shown in Table 3, the new method obtained the minimum value in the average absolute error of all samples compared with other methods, which indicated the diameters of trees fitted by this method were accurately close to the observed diameter. Similarly, the max error of radius with this algorithm was limited into 9 mm in different positions and distances. This parameter demonstrated that, when the data points lay along a circular arc with low curvature, the new method avoided catastrophic cancelations of large circles with a large radius and a far away center such as TDE. Similarly, the average relative radius error was following a diminishing trend during the experiment concerned and reached the minimum value by using the new algorithm, and was decreased by 44.9% compared with the best of the other methods, CFAA-MS. This meant that the new algorithm was suitable for different test constraints with totally chaotic samples and was more stable than others.

For testing the measurement effectiveness of each algorithm further, the proposed algorithm was compared with a few others in a series of statistic parameters. The Root Mean Square error (RMSE) of the absolute errors of estimated diameter in this algorithm was close to 4.5 mm, which was the smallest among all methods. The value of RMSE in this paper showed the obvious improvement by 71.5% compared with TDE and by 30.9% corresponding to CFAA-MS, which suggested a higher fitting precision of this chosen estimated model and a better prediction ability for laser data. What’s more, the minimum value of the Standard Deviation (STD) for the estimated diameter errors obtained by this proposed method indicated that the error distribution was not very discrete. In a sense, this measuring system could deal with the worst case scenario corresponding to very noisy laser data. Thus it indicated that this algorithm was suitable for the most challenging conditions and was more stable and robust than others in this paper. 

Next, the coefficient of determination of regression squares (R-square) was applied to demonstrate the superiority of our new algorithm over the main existing algorithms. This parameter was decided by the sum of squares of the regression (SSR) divided by the total sum of squares (SST), which was through the change of the data to represent the fitting effect. By the above expression, the normal value of R-square was distributed in the certain range of [0, 1]. The numerical result in this new algorithm obtained a maximal value approximating 1, which suggested that the equation of this circle fitting method had stronger diameter estimation ability compared with the others. Also the estimation errors were weakly affected by distances and poses of the object and the fitting result for diameters was more stable and accurate. Lastly, Table 3 gives the time consumption for the calculation by different algorithms, respectively. Except for the TDE method, the new method displayed the minimum time consumption, which was reduced enormously compared with the F-R and PRP algorithms. This time consumption of the calculation met the requirements of the accuracy and real time for logging harvesting operations. 

A further experiment dealing with the influencing factors on the estimation error was performed. The errors were mainly caused by the resolution, specular energy errors of the laser device and the approximation errors of the fitting algorithm. The error also was impacted by the distance between the base point of the laser scanner and the device. In addition, the distance was positively related to the size of the circles corresponding to the actual diameter of the trees. To confirm the significant factors, the paper was simplified with removing insignificant factors if they did not have any significant main or interaction effect. Then the iterations, the tree trunk diameter and its distance from the scanner were entered as core-variates affecting the error, which would be analyzed further.

### 4.3. Error Factor Discussion

To reveal the influence of the number of repeated laser scans on the absolute error of diameter estimation, an experiment was performed with the abovementioned trees 1 to 8. Those birches were encircled with coarse white bark at a height of 1.3 m above the ground, which made the reflectivity of scanned trees increase up to 100%. This eliminated the influence of different reflectivity on the diameter estimation. Then the 2DTLS acquired the laser scanning data of all trees for 100 consecutive trials. Consequently, the point clouds were distributed around the outline of trunk at a slight difference, which was caused by the fluctuating error as seen in the abovementioned analysis. According to Equation (7), the target clusters scanned at different times were applied to form an optimized cluster, which represented the mean value of several scans. For each tree, the number of multiple scans changed from 1 to 100, which was defined as the repetition number. As a consequence, there were 100 optimized laser data being generated to calculate the absolute errors for DBH estimation of one tree.

**Figure 10:**
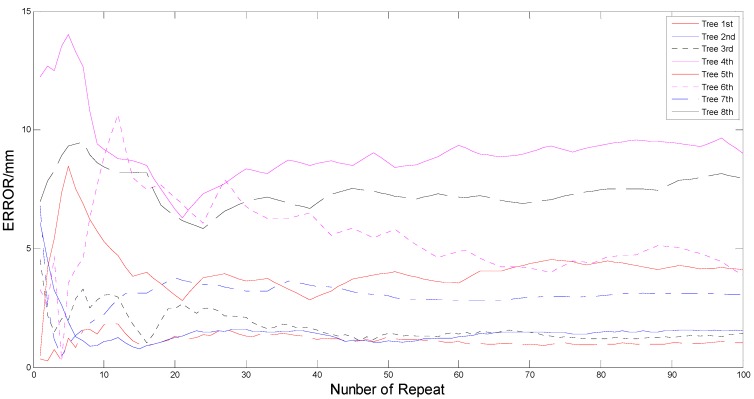
The relationship between the number of repeat and the absolute error of diameter estimation for all trees.

The variation tendency between the absolute estimation error and the number of repeat is displayed in Figure 10, where the *x*-axis represented the number of repeats in the range of 1 to 100, and the *y*-axis represented the estimation error for diameters. When analyzing the effects on all targets in general, it is revealed that the absolute errors of most trees were increased at the earlier stage, then decreased and finally tended to be stable with increasing number of repeats. The relationship is statistically significant except for the second tree. The error of the second tree decreased from the start until 8 and tended to smooth with the minimum value at 15. 

A specific section analysis of the others suggested that the errors increased to the peaks of the curve at different number. Trees 1, 3 and 6 gave a maximum error value between 11 and 13, while the other trees (4, 5 and 8) reached the peak value in the range of 6 to 8. Only the 7th tree gave the maximum at 20. Then, all curves of error and repeated number decreased to the smallest error threshold at a similar range between 20 and 25, including the second tree. In spite of different changes (Tree 4 and 8 rose, but the trees 1, 3, 5 and 7 fluctuated over a small range), the error curves tended to smooth after about 45 except for the 6th tree, which continued to decline until 70 and then tended to be stable, so according to the experimental results, it could be seen that 20 repeats had reduced the laser fluctuating error, which would effectively improve the accuracy of diameter estimation. As a result of considering that the scanning frequency of 2DTLS was set as 100 Hz, taking 20 scans to calculate the average just consumed 0.2 s in measuring process, which met the requirements of the real-time and accuracy for the measuring system in forestry harvesters. Therefore, it could be concluded that the design of this repeat number was optimal in terms of minimization of estimated errors and the actual forestry vehicle applications.

**Figure 11:**
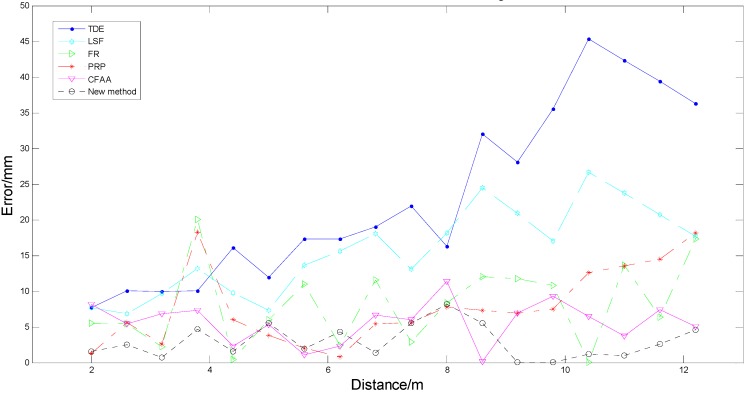
The changes in the relationship between the distance and the estimation error.

However, the Figure 10 also revealed that the errors of diameter estimation were very different for different targets, which had diverse diameters and were located at different distances from the 2DTLS. Therefore, a further outdoor experiment was performed to reveal the influence of the distance on the average absolute error of diameter estimation. For each abovementioned birch, the 2DTLS was placed at a distance ranging from 2 m to 12.2 m to the tree in 0.6 m steps. Then the device scanned all eight targets 20 times to achieve the optimized laser data. In order to increase the quantity of targets, thirteen tree trunk sections with diameters in the range of 9–35 cm and lengths in the range of 40–49 cm were used for the indoor experiment as recorded in Table 1. Considering that the measured trees in outdoor experiment were all silver birches, the distinguishing species were placed in an indoor corridor, at distances varying between 2 m and 12.2 m every 0.6 m, with varying sides facing the laser scanner. Then the 2DTLS was set at the same height of 35 cm above the ground and all observations were scanned 20 times in various combinations. In total, 378 sets of laser data integrating the outside and indoor measurement experiments were scanned to calculate the diameter estimation errors for a better statistical determination. When analyzing the effects on one treatment combination (distance-error) in the proposed methods, the laser data at same distance (21 sets every distance) were applied to compute the fitting diameters in all the methods mentioned above. For each algorithm, the average of diameter estimation errors in one distance was performed to draw the distance-error curve as illustrated in Figure 11, where the *x*-axis represented the distance between the trees and the laser scanner, and the *y*-axis represented the average of estimated errors. 

A simple analysis explained that the trend of the average estimation error was increasing with increasing distance for most methods. The error curve using the TDE and LSF algorithms rose persistently with increasing distances in the distance range, as well as the curve computed with the PRP method except for the sudden huge error at some distances (3.8 m). For the FR method, the error was badly disturbed by noise and did not increase with distance until the end of the curve, but the relationship only explained 66.7% of the observed variation respectively except for the results calculated by the CFAA and the new algorithms. The new algorithm and the CFAA method had less change error with increasing the distance, in other words, the errors obtained by the two methods were affected weakly by the distance. The error curve of this proposed algorithm was smoother than the CFAA curve and had smaller values for most distances, which showed the higher accuracy and stability in estimating the diameters of trees with this algorithm. Therefore, this proposed method was suitable for the measuring tasks of the logging harvester operations when the cutting targets were distributed at different distances.

**Figure 12:**
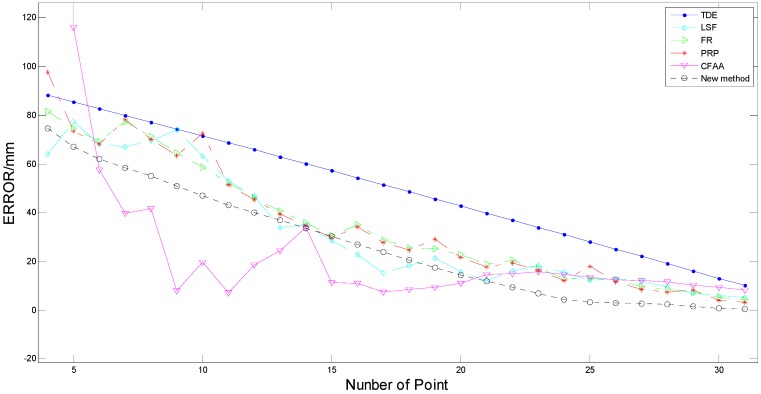
The estimation error decreased with increasing number of point hitting the tree, which was associated with the size of the circles.

In view of that the error curves was seriously noisy, a potential cause was that the average error in one distance was achieved by using tree trunks with different diameters. Thereby, a depth analysis was carried out to reveal the effects of diameter on the estimation error. To avoid the influence of distance on the error, only partial laser data at three distances (2 m, 2.6 m and 3.2 m) were chosen as independent observations. Meanwhile, the number of laser points hitting the tree corresponded to the actual diameter of the trees and distances. Thus, the parameter was designed as a single predictor to reveal the influence of the trunk diameters on the estimated errors. Finally, 29 sets of data were randomly selected to compute the estimation errors in different algorithms as shown in Figure 12. For observation trees with diameters between 9 and 35 cm, there was a reasonable relationship in that the error decreased with increasing tree trunk diameter in different positions and distances. As presented in Figure 12, the result demonstrates that the estimation error was influenced by tree trunk diameter in a negative correlation. The error curve in the proposed algorithm was much smoother than the curves in other methods except for the TDE algorithm, which obtained an even smoother (almost linear) curve. This indicated that the new method lowered the error noise regardless of the number of points, and in a sense, this method was much more stable in the process of estimating diameter errors corresponding to most of the other methods. Moreover, the new method obtained the lowest error value comparing with most of the algorithms in the range between 3 and 21, except for the CFAA algorithm, which was affected significantly by the noise. When the number of points reached 21, the proposed method maintained the minimum value. Thus, with the efficiency of the diameters varying between distances, our algorithm could have highest accuracy in estimating the diameters of the tree trunks and suffered lower error estimation noise.

## 5. Conclusions and Outlook

In summary, a new algorithm to improve the accuracy of tree trunk diameter estimating in forest area is proposed in this paper. First, the measuring information is collected by laser using a 2D laser scanner and an infrared thermal imager. Then, after cluster extraction and filtration, the features of the trunk could be obtained from the raw laser point cloud. Further, by optimizing the laser data based on the arithmetic mean method, a new hybrid algorithm based on an algebraic circle fitting algorithm in polar form fused with a non-linear optimization principle in the Levenberg-Marquardt method is generally used to determine the radii and positions of the trees.

Compared with previous works published by other researchers, the experimental results show that the proposed measuring system accomplishes the trunk detection and diameter estimation of trees effectively with the minimum value in the average absolute error and average relative error, which indicates that the estimated diameters best fitted the observed diameter. Moreover, by analyzing the RMSE, STD and R-square, we found that this proposed method is suitable for the most challenging conditions and is more stable and robust than others while also showing reduced calculation times, which are practical significance in improving the operating efficiency of forest harvesters and reducing the risks of causing accidents.

Finally, this paper reveals the influence of the number of repeats on the estimation error. The experimental results indicate 20 times is the best value of this repeat number, which will reduce the laser fluctuation errors and effectively improve the accuracy of diameter estimation. Furthermore, according to our study of the effects of external factors (diameters and distances) on the estimation error, the hybrid algorithm performs well in improving the estimation effectiveness of tree trunk diameter. Thus the improved diameter estimation algorithm is important for forestry logging operations, localization and automation of forest machines, SLAM generation of local maps and so on. However, the current diameter estimation method is proposed on the basis of a static system. In the future, a dynamic diameter estimation combining a laser scanner with cameras will be studied in order to achieve real-time and rapid measuring results in the forest environment.
